# Editorial: Molecular Targets and Therapeutic Strategies in Gynecological Cancers

**DOI:** 10.3389/fonc.2022.904032

**Published:** 2022-04-14

**Authors:** Ziwen Lu, Hanqing Liu

**Affiliations:** School of Pharmacy, Jiangsu University, Zhenjiang, China

**Keywords:** gynecological cancers, vascular mimicry (VM), HIF-1α, quercetin, cisplatin, *ex vivo* model, molecular targets, tumor microenvironment

Gynecological malignancies, including cancers originating from the ovaries, uterus, cervix, vagina, fallopian tubes, and vulva, are global health threat for women. Although these cancers often have different clinicopathological characteristics, histopathological traits, as well as treatment strategies, similar pathological signaling pathways and oncogenic signaling hubs, such as many DNA damage response-related proteins, cell-cycle and apoptosis regulators, stimulators of angiogenesis, pro-angiogenic proteins, as well as inflammation-associated cytokines, play pivotal roles in the occurrence and development of these cancers. Despite surgical, radiotherapeutic, chemotherapeutic, and targeted therapeutic advances in the past two decades, effective therapeutic strategies for gynecological cancers remain challenging. This Research Topic aims at providing updates on the newly identified molecular targets and developed therapeutic strategies for gynecological cancers, and discussing the recent advances on screening, diagnosis, therapies, and prognosis of gynecological cancers.

Growing evidence indicates that initiation, progression and therapeutic response are strongly influenced by paracellular signaling between the cancer cells and tumor microenvironment (TME) (1). Lack of treatment strategies effectively treating or curing gynecological cancers has sparked interest in the investigation of the roles of the TME in the pathogenesis of these cancers, which may provide insights into identifying novel targets and developing drugs against these targets. Since the introduction of vascular mimicry (VM) in melanoma, defined as perfusable, matrix-rich, and vasculogenic-like networks by deregulated cancer cells in 3-D matrices *in vitro* paralleling matrix-rich networks in patients’ aggressive tumors, VM has been characterized in carcinomas of ovary, breast, and other cancers (2–4). In this Research Topic, Charfi et al. described a couple of novel doxorubicin-peptide and docetaxel-peptide conjugates which significantly decreased *in vitro* VM of both TNBC-derived MDA-MB-231 breast and ovarian ES-2 clear cell carcinoma cells at low nM or even pM concentrations, in addition to their reported cytotoxicity. They also demonstrated that expression of sortilin (SORT1) is required for *in vitro* VM and is associated with VM in tumor xenografts, and inhibition of the peptide conjugates requires SORT1. As we know, hypoxia and activation of its main effector, hypoxia-inducible factor-1 (HIF-1) are typical for advanced solid tumors (5, 6). In addition, hypoxia often induces a plethora of events in the TME and facilitates crosstalk between tumor cells and nonmalignant cells. Wang et al. focused on the HIF-1α as a potential target for ovarian cancer therapies in a review of this Research Topic. They discussed cancer progression-associated signaling pathways affected by HIF-1α, such as p53, IL-6, AKT/mTOR, and glycolysis pathways. They also summarized interaction and crosstalk mediated *via* HIF-1α between cancer cells and mesothelial cells, immune cells, and adipocytes. In addition, current clinical trials targeting HIF-1α and molecules directly or indirectly suppressing ovarian cancer progression by downregulating HIF-1α were also discussed.

Recently, advances in systems biology, network pharmacology, and polypharmacology have inspired studies in developing novel multi-targeted drugs from global traditional medicine for optimal efficacy and tolerable toxicity. Ji et al. predicted potentially synergistic targets for cisplatin treatment *via* overlapping target genes of cisplatin, cervical cancer, and Yimucao, a Chinese traditional medicine. In addition, quercetin was identified as the top active compound of Yimucao by compound-target network, and the anticancer effects and signaling pathways of quercetin enhancing the cytotoxicities of cisplatin in cervical cancer cells was predicted and verified.

In another original research article in this Research Topic, Zhang et al. retrospectively analyzed the data and tissue specimens of patients with clear cell carcinoma (CCC) of the endometrium, a rare and invasive tumor, and found that age at the diagnosis, FIGO stage, tumor size, myometrial infiltration, Ki-67 index, positive expression of P53, lymphovascular invasion, and distant metastasis were significantly associated with shorter overall survival. In addition, CCC has a significantly worse prognosis than endometrioid carcinoma other than uterine papillary serous carcinoma.

In the current era of personalized medicine, due to the lack of reliable preclinical disease or “near-patient” models, extraordinarily high heterogeneity of gynecological malignancies, and interactions between the cancer cells and the cellular and acellular TME, novel *ex vivo* systems to expedite research and development of anticancer drugs, guide clinical decision-making, and offer timing advantages for prospective personalized models are in a great demand. Clark et al. discussed the advantages, limitations, and potential applications of current 3D ovarian cancer models, especially spheroids, patient derived organoids (PODs), patient derived xenografts (PDX), tumor slices/explants, and organotypic omental models. They also reviewed novel technologies in 3D models, such as microfluidic based models, models with alternative extracellular matrices, and models with *in vitro* incorporation of functioning TME.

Overall, the collections of research articles and reviews under this Research Topic will hopefully encourage us to explore molecular targets from various perspectives and stimulate critical evaluation of novel therapeutic strategies in gynecological cancers.

## Author Contributions

All authors listed have made a substantial, direct and intellectual contribution to the work, and approved it for publication.

## Funding

This work was supported by the National Natural Science Foundation 81402145, 81672582 (to HL); Top Talent of Innovative Research Team of Jiangsu Province (to HL).

## Conflict of Interest

The authors declare that the research was conducted in the absence of any commercial or financial relationships that could be construed as a potential conflict of interest.

## Publisher’s Note

All claims expressed in this article are solely those of the authors and do not necessarily represent those of their affiliated organizations, or those of the publisher, the editors and the reviewers. Any product that may be evaluated in this article, or claim that may be made by its manufacturer, is not guaranteed or endorsed by the publisher.
